# Crystal structure of anhydrous poly[bis­(μ_2_-sarcosinato-κ^3^ *O*,*N*:*O*′)copper(II)]

**DOI:** 10.1107/S1600536814020418

**Published:** 2014-09-17

**Authors:** Ray J. Butcher, Greg Brewer, Matthew Zemba

**Affiliations:** a Department of Chemistry, Howard University, 525 College Street NW, Washington, DC 20059, USA; b Department of Chemistry, The Catholic University of America, 620 Michigan Ave., N.E., Washington, DC 20064, USA

**Keywords:** crystal structure, anhydrous bis­(sarcosinato)copper(II), non-proteinogenic amino acid

## Abstract

The title compound, [Cu(C_3_H_6_NO_2_)_2_]_*n*_, is a bis-complex of the anion of sarcosine (*N*-methyl­glycine). The asymmetric unit consists of a copper(II) ion, located on a center of inversion, and one mol­ecule of the uninegative sarcosinate anion. The copper(II) ion exhibits a typical Jahn–Teller distorted [4 + 2] coordination geometry. The four shorter equatorial bonds are to the nitro­gen and carboxyl­ate O atoms of two sarcosinate anions, and the longer axial bonds are to carboxyl­ate O atoms of neighboring complexes. The overall structure is made up from two chains formed by these longer axial Cu—O bonds, one extending parallel to [011] and the other parallel to [0-11]. Each one-dimensional array is connected by the equatorial bridging moieties to the chains on either side, creating an extended two-dimensional framework parallel to (100). There is a single inter­molecular hydrogen-bonding inter­action within the sheets between the amino NH group and an O atom of an adjacent mol­ecule.

## Abstract 2

The copper(II) ion of the anhydrous form of bis­(sarcosinato)copper(II) exhibits a [4 + 2] coordination sphere with four shorter equatorial bonds to the N and carboxyl­ate O atoms of two sarcosinate anions, and two longer axial bonds to O atoms of neighboring complexes, leading to a sheet structure parallel to (001).

## Chemical context   

The α-amino acids are essential for life as they are the building blocks of all proteins and enzymes and a great deal is known about their structures and complexes. *N*-Methyl amino acids, such as sarcosine, are non-proteinogenic and hence differ from the proteinogenic amino acids used in living systems in that the amino N atom is achiral in the free mol­ecule but chiral, *R* or *S*, when bound to a metal. Examples of complexes of sarcosine that exhibit chirality due to coordination of the amino N atom have been reported (Blount *et al.*, 1967 ▶; Larsen *et al.*, 1968 ▶; Prout *et al.*, 1972 ▶). This is similar to the chirality that is observed on the binding of reduced tripodal Schiff base complexes of metals (Brewer *et al.*, 2014 ▶; Al-Obaidi *et al.*, 1996 ▶). In these cases, the binding of three achiral (due to rapid inversion) amine N atoms of the free ligand to the same metal resulted in the observation of a single enanti­omeric pair (*RRR* and *SSS*) or a single enanti­omer (*RRR* or *SSS*) if the mol­ecule crystallized in one of the Sohncke space groups. In these cases, the binding of an organic ligand containing three achiral N atoms to a metal resulted in a preference for chirality correlation of the N atoms, *RRR* or *SSS*, resulting in homochiral complexes. Similarly, the reduced Schiff base complexes of the condensate of amino acids with salicyl­aldehyde have an energetic preference for the stereoisomer in which the chirality of the α-C atom and the amine N atom are correlated (Koh *et al.*, 1996 ▶).
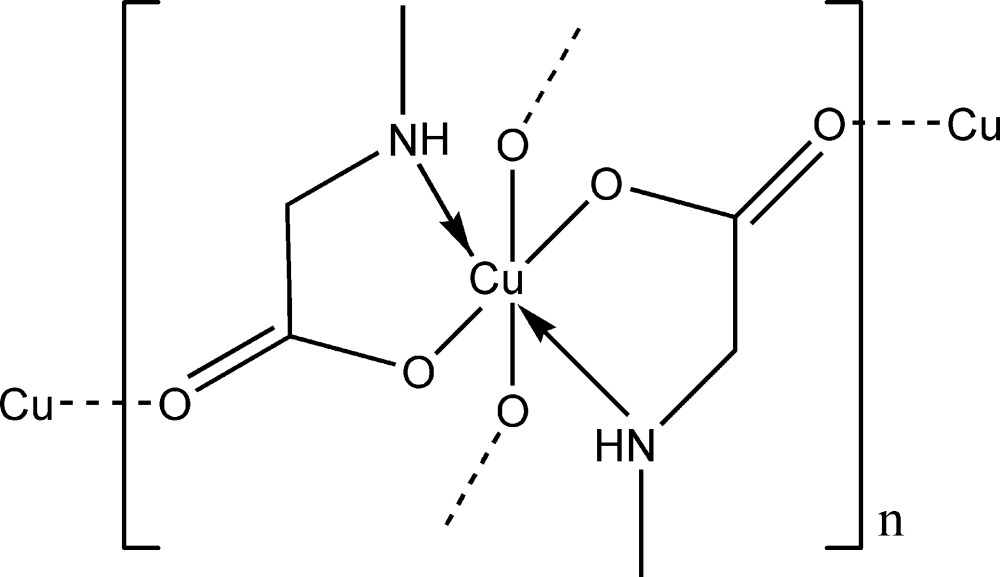


This preference for homochirality is not always observed: the copper complex of a Schiff base condensate of tyrosine is heterochiral as is the bis-adduct of cobalt(III) with histidine (Pradeep *et al.*, 2006 ▶; Zie *et al.*, 2007 ▶). The present complex was investigated to determine if there was a preference for homo- (*RR* or *SS*) *versus* heterochirality (*RS*) in a *M*(sarcosinato)_2_ complex (*M* = divalent transition metal). Heterochirality, *RS*, was observed in this complex. Future work will focus on related complexes such as *M*′(sarcosinato)_3_ (*M*′ = trivalent transition metal) to determine if the presence of three chiral ligands bound to a single metal favors homochirality, which can serve as a method of enanti­omeric separation.

## Structural commentary   

**Figure 1:**
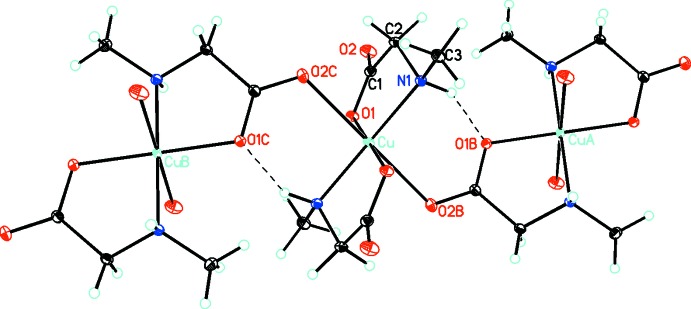
Part of a chain in the title compound, with the atom-numbering scheme and atomic displacement parameters drawn at the 30% probability level. Hydrogen bonding is shown by dashed lines. [Symmetry codes: (A) 1 − *x*, 1 − *y*, 1 − *z*; (B) 1 − *x*, *y* − 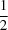, 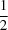 − *z*; (C) *x*, 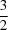 − *y*, 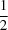 + *z*.]

The title compound, [Cu(C_3_H_6_NO_2_)_2_]_*n*_, is a bis-complex of the sarcosinate anion with copper(II). The central metal cation is located on a center of inversion. It is six-coordinate and has a distorted octa­hedral [4 + 2] coordination sphere characteristic for Jahn–Teller systems. The four shorter equatorial bonds are to the amino N atom and carboxyl­ate O atom of two sarcosinate anions (Fig. 1 ▶). The N and O atoms are *trans* to one another. The related [Cu(sarcosinato)_2_]·2H_2_O structure (Krishnakumar *et al.*, 1994 ▶) is much simpler in that the two longer axial bonds are to water mol­ecules so that there is no extended bonding to neighboring complexes. In both structures, the equatorial Cu—O and Cu—N bond lengths are very similar [Cu—O = 1.9758 (8) Å and Cu—N = 2.0046 (9) Å in the title compound, and 1.970 and 2.007 Å in the dihydrate], but the axial Cu—O distances are significantly different at 2.5451 (10) and 2.461 Å.

## Supra­molecular features   

**Figure 2:**
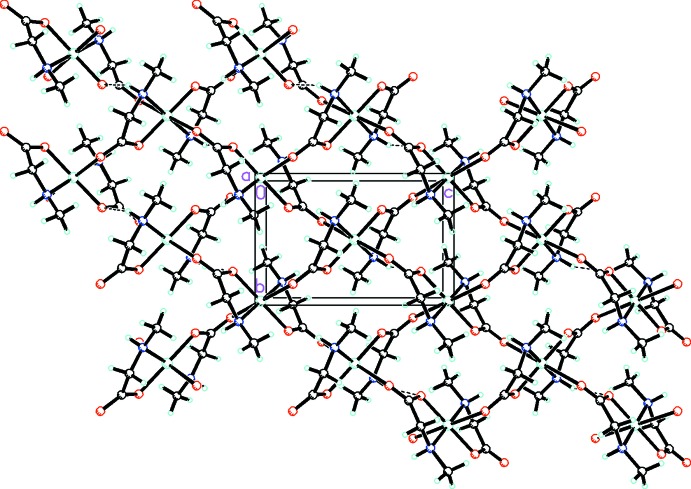
Packing diagram of the title compound viewed along the *a* axis. Hydrogen bonding is shown by dashed lines.

In the title compound, the individual coordination polyhedra are linked by longer axial Cu—O bonds into two chains, one extending parallel to [011] and the other parallel to [0
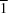
1]. The one-dimensional array is linked by equatorial bridging bonds to the chains on either side, creating an extended two-dimensional framework (Fig. 2 ▶) parallel to (100). There is a single inter­molecular hydrogen-bonding inter­action within the sheets between the amino NH and an carboxyl­ate O atom of an adjacent mol­ecule (Table 1 ▶).

## Database survey   

The structure of the zwitterionic form of sarcosine has been reported by Rodrigues *et al.* (2005 ▶). The structure of the copper(II) and nickel(II) complexes of this same ligand have been reported as their dihydrates by Krishnakumar *et al.* (1994 ▶) and Guha (1973 ▶), respectively.

## Synthesis and crystallization   

Sarcosine (*N*-methyl­glycine) was purchased from Aldrich Chemical. Sarcosine (1.87 mmol, 0.166 g) was dissolved in 0.10 *M* aqueous potassium hydroxide (18.7 ml, 1.87 mmol). Copper chloride dihydrate (0.468 mmol, 0.078 g) was added to the above solution and crystals of the title compound were grown by slow evaporation.

## Refinement   

Crystal data, data collection and structure refinement details are summarized in Table 2 ▶. H atoms were positioned geom­etrically and refined using a riding model, with C—H distances of 0.93–0.99 Å, and with *U*_iso_(H) = 1.2*U*_eq_(C) or 1.5*U*_eq_(C) for methyl H atoms. The H attached to N was located in a difference Fourier map and refined using a riding model, with an N—H distance of 0.93 Å and *U_iso_*(H) = 1.2*U_eq_*(N).

## Supplementary Material

Crystal structure: contains datablock(s) I. DOI: 10.1107/S1600536814020418/wm5056sup1.cif

Structure factors: contains datablock(s) I. DOI: 10.1107/S1600536814020418/wm5056Isup2.hkl

CCDC reference: 961026

Additional supporting information:  crystallographic information; 3D view; checkCIF report

## Figures and Tables

**Table 1:** Hydrogen-bond geometry (Å, °)

*D*—H⋯*A*	*D*—H	H⋯*A*	*D*⋯*A*	*D*—H⋯*A*
N1—H1*A*⋯O1^i^	0.93	2.13	2.9729 (13)	150

Symmetry code: (i) 
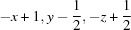
.

**Table 2:** Experimental details

Crystal data	
Chemical formula	[Cu(C_3_H_6_NO_2_)_2_]
*M*_r_	239.72
Crystal system, space group	Monoclinic, *P*2_1_/*c*
Temperature (K)	123
*a*, *b*, *c* (Å)	7.9031 (3), 5.9461 (2), 8.9907 (3)
β (°)	90.039 (3)
*V* (Å^3^)	422.50 (3)
*Z*	2
Radiation type	Mo *K*α
μ (mm^−1^)	2.57
Crystal size (mm)	0.51 × 0.45 × 0.12

Data collection	
Diffractometer	Agilent Xcalibur Ruby Gemini
Absorption correction	Multi-scan (*CrysAlis PRO*; Agilent, 2012 ▶)
*T*_min_, *T*_max_	0.478, 1.000
No. of measured, independent and observed [*I* > 2σ(*I*)] reflections	5479, 1768, 1542
*R*_int_	0.025
(sin θ/λ)_max_ (Å^−1^)	0.808

Refinement	
*R*[*F* ^2^ > 2σ(*F* ^2^)], *wR*(*F* ^2^), *S*	0.023, 0.061, 1.08
No. of reflections	1768
No. of parameters	63
H-atom treatment	H-atom parameters constrained
Δρ_max_, Δρ_min_ (e Å^−3^)	0.64, −0.36

Computer programs: *CrysAlis PRO* (Agilent, 2012 ▶), *SIR97* (Altomare *et al.*, 1999 ▶), *SHELXL97* and *SHELXTL* (Sheldrick, 2008 ▶).

## supplementary crystallographic information

### Crystal data

**Table:**

[Cu(C_3_H_6_NO_2_)_2_]	*F*(000) = 246
*M**_r_* = 239.72	*D*_x_ = 1.884 Mg m^−^^3^
Monoclinic, *P*2_1_/*c*	Mo *K*α radiation, λ = 0.71073 Å
Hall symbol: -P 2ybc	Cell parameters from 2393 reflections
*a* = 7.9031 (3) Å	θ = 3.4–35.0°
*b* = 5.9461 (2) Å	µ = 2.57 mm^−^^1^
*c* = 8.9907 (3) Å	*T* = 123 K
β = 90.039 (3)°	Plate, dark blue
*V* = 422.50 (3) Å^3^	0.51 × 0.45 × 0.12 mm
*Z* = 2	

### Data collection

**Table:**

Agilent Xcalibur Ruby Gemini diffractometer	1768 independent reflections
Radiation source: Enhance (Mo) X-ray Source	1542 reflections with *I* > 2σ(*I*)
Graphite monochromator	*R*_int_ = 0.025
Detector resolution: 10.5081 pixels mm^-1^	θ_max_ = 35.0°, θ_min_ = 4.1°
ω scans	*h* = −12→11
Absorption correction: multi-scan (*CrysAlis PRO*; Agilent, 2012)	*k* = −9→8
*T*_min_ = 0.478, *T*_max_ = 1.000	*l* = −14→14
5479 measured reflections	

### Refinement

**Table:**

Refinement on *F*^2^	Secondary atom site location: difference Fourier map
Least-squares matrix: full	Hydrogen site location: inferred from neighbouring sites
*R*[*F*^2^ > 2σ(*F*^2^)] = 0.023	H-atom parameters constrained
*wR*(*F*^2^) = 0.061	*w* = 1/[σ^2^(*F*_o_^2^) + (0.0285*P*)^2^ + 0.0835*P*] where *P* = (*F*_o_^2^ + 2*F*_c_^2^)/3
*S* = 1.08	(Δ/σ)_max_ < 0.001
1768 reflections	Δρ_max_ = 0.64 e Å^−^^3^
63 parameters	Δρ_min_ = −0.36 e Å^−^^3^
0 restraints	Extinction correction: *SHELXTL* (Sheldrick, 2008), Fc^*^=kFc[1+0.001xFc^2^λ^3^/sin(2θ)]^-1/4^
Primary atom site location: structure-invariant direct methods	Extinction coefficient: 0.014 (3)

### Special details

**Table:**

Geometry. All e.s.d.'s (except the e.s.d. in the dihedral angle between two l.s. planes) are estimated using the full covariance matrix. The cell e.s.d.'s are taken into account individually in the estimation of e.s.d.'s in distances, angles and torsion angles; correlations between e.s.d.'s in cell parameters are only used when they are defined by crystal symmetry. An approximate (isotropic) treatment of cell e.s.d.'s is used for estimating e.s.d.'s involving l.s. planes.
Refinement. Refinement of *F*^2^ against ALL reflections. The weighted *R*-factor *wR* and goodness of fit *S* are based on *F*^2^, conventional *R*-factors *R* are based on *F*, with *F* set to zero for negative *F*^2^. The threshold expression of *F*^2^ > σ(*F*^2^) is used only for calculating *R*-factors(gt) *etc*. and is not relevant to the choice of reflections for refinement. *R*-factors based on *F*^2^ are statistically about twice as large as those based on *F*, and *R*- factors based on ALL data will be even larger.

### Fractional atomic coordinates and isotropic or equivalent isotropic displacement parameters (Å^2^)

**Table:**

	*x*	*y*	*z*	*U*_iso_*/*U*_eq_	
Cu	0.5000	0.5000	0.5000	0.01076 (7)	
O1	0.53710 (10)	0.74236 (14)	0.35326 (9)	0.01390 (16)	
O2	0.72233 (12)	0.85036 (16)	0.17943 (10)	0.0221 (2)	
N1	0.69539 (12)	0.35314 (16)	0.39628 (10)	0.01218 (17)	
H1A	0.6524	0.2775	0.3140	0.015*	
C1	0.67866 (14)	0.72698 (19)	0.28370 (12)	0.01373 (19)	
C2	0.79685 (14)	0.5444 (2)	0.34046 (13)	0.0135 (2)	
H2A	0.8718	0.4933	0.2590	0.016*	
H2B	0.8685	0.6048	0.4215	0.016*	
C3	0.79798 (15)	0.1919 (2)	0.48149 (14)	0.0171 (2)	
H3A	0.8941	0.1425	0.4209	0.026*	
H3B	0.7284	0.0615	0.5080	0.026*	
H3C	0.8398	0.2642	0.5723	0.026*	

### Atomic displacement parameters (Å^2^)

**Table:**

	*U*^11^	*U*^22^	*U*^33^	*U*^12^	*U*^13^	*U*^23^
Cu	0.00955 (10)	0.01254 (10)	0.01019 (10)	0.00153 (6)	0.00211 (6)	0.00253 (6)
O1	0.0127 (3)	0.0158 (4)	0.0132 (3)	0.0014 (3)	0.0018 (3)	0.0034 (3)
O2	0.0215 (4)	0.0249 (5)	0.0199 (4)	0.0014 (4)	0.0060 (3)	0.0105 (4)
N1	0.0111 (4)	0.0142 (4)	0.0112 (4)	−0.0001 (3)	0.0001 (3)	0.0001 (3)
C1	0.0132 (4)	0.0152 (5)	0.0128 (4)	−0.0016 (4)	0.0005 (4)	0.0008 (4)
C2	0.0107 (4)	0.0165 (5)	0.0132 (5)	−0.0008 (4)	0.0011 (4)	0.0012 (4)
C3	0.0161 (5)	0.0168 (5)	0.0183 (5)	0.0040 (4)	0.0003 (4)	0.0020 (4)

### Geometric parameters (Å, º)

**Table:**

Cu—O1^i^	1.9758 (8)	N1—C2	1.4796 (15)
Cu—O1	1.9758 (8)	N1—H1A	0.9300
Cu—N1^i^	2.0046 (9)	C1—C2	1.5200 (17)
Cu—N1	2.0046 (9)	C2—H2A	0.9900
Cu—O2^ii^	2.5451 (10)	C2—H2B	0.9900
Cu—O2^iii^	2.5451 (10)	C3—H3A	0.9800
O1—C1	1.2853 (13)	C3—H3B	0.9800
O2—C1	1.2396 (14)	C3—H3C	0.9800
N1—C3	1.4705 (15)		
			
O1^i^—Cu—O1	180.0	C3—N1—H1A	107.4
O1^i^—Cu—N1^i^	83.82 (3)	C2—N1—H1A	107.4
O1—Cu—N1^i^	96.18 (3)	Cu—N1—H1A	107.4
O1^i^—Cu—N1	96.18 (3)	O2—C1—O1	124.66 (11)
O1—Cu—N1	83.82 (3)	O2—C1—C2	120.34 (10)
N1^i^—Cu—N1	180.0	O1—C1—C2	114.98 (10)
O1^i^—Cu—O2^ii^	86.24 (3)	N1—C2—C1	109.26 (9)
O1—Cu—O2^ii^	93.76 (3)	N1—C2—H2A	109.8
N1^i^—Cu—O2^ii^	94.85 (3)	C1—C2—H2A	109.8
N1—Cu—O2^ii^	85.15 (3)	N1—C2—H2B	109.8
O1^i^—Cu—O2^iii^	93.76 (3)	C1—C2—H2B	109.8
O1—Cu—O2^iii^	86.24 (3)	H2A—C2—H2B	108.3
N1^i^—Cu—O2^iii^	85.15 (3)	N1—C3—H3A	109.5
N1—Cu—O2^iii^	94.85 (3)	N1—C3—H3B	109.5
O2^ii^—Cu—O2^iii^	180.0	H3A—C3—H3B	109.5
C1—O1—Cu	113.74 (7)	N1—C3—H3C	109.5
C3—N1—C2	112.28 (9)	H3A—C3—H3C	109.5
C3—N1—Cu	117.79 (7)	H3B—C3—H3C	109.5
C2—N1—Cu	103.93 (7)		
			
O1^i^—Cu—O1—C1	−64 (100)	O1—Cu—N1—C2	29.24 (7)
N1^i^—Cu—O1—C1	166.53 (8)	N1^i^—Cu—N1—C2	80 (100)
N1—Cu—O1—C1	−13.47 (8)	O2^ii^—Cu—N1—C2	−65.08 (7)
O2^ii^—Cu—O1—C1	71.23 (8)	O2^iii^—Cu—N1—C2	114.92 (7)
O2^iii^—Cu—O1—C1	−108.77 (8)	Cu—O1—C1—O2	174.29 (10)
O1^i^—Cu—N1—C3	−25.85 (8)	Cu—O1—C1—C2	−6.96 (12)
O1—Cu—N1—C3	154.15 (8)	C3—N1—C2—C1	−167.81 (9)
N1^i^—Cu—N1—C3	−155 (100)	Cu—N1—C2—C1	−39.44 (10)
O2^ii^—Cu—N1—C3	59.83 (8)	O2—C1—C2—N1	−148.69 (11)
O2^iii^—Cu—N1—C3	−120.17 (8)	O1—C1—C2—N1	32.50 (13)
O1^i^—Cu—N1—C2	−150.76 (7)		

Symmetry codes: (i) −*x*+1, −*y*+1, −*z*+1; (ii) *x*, −*y*+3/2, *z*+1/2; (iii) −*x*+1, *y*−1/2, −*z*+1/2.

### Hydrogen-bond geometry (Å, º)

**Table:**

*D*—H···*A*	*D*—H	H···*A*	*D*···*A*	*D*—H···*A*
N1—H1*A*···O1^iii^	0.93	2.13	2.9729 (13)	150

Symmetry code: (iii) −*x*+1, *y*−1/2, −*z*+1/2.
